# Genome sequence and comparative analysis of a *Vibrio cholerae* O139 strain E306 isolated from a cholera case in China

**DOI:** 10.1186/1757-4749-6-3

**Published:** 2014-02-12

**Authors:** Yong Yi, Na Lu, Fei Liu, Jing Li, Ruifen Zhang, Liping Jia, Hua Jing, Hu Xia, Yi Yang, Baoli Zhu, Yongfei Hu, Yan Cui

**Affiliations:** 1 The 306th Hospital of PLA, Beijing 100101, China; 2 CAS key Laboratory of Pathogenic Microbiology and Immunology, Institute of Microbiology, Chinese Academy of Sciences, Beijing 100101, China; 3 Beijing Key Laboratory of Microbial Drug Resistance and Resistome, Beijing 100101, China; 4 Department of Obstetrics and Gynecology, Peking Union Medical College Hospital, Peking Union Medical College and Chinese Academy of Medical Sciences, Beijing 100730, China

**Keywords:** Cholera toxin prophage, Integrative conjugative elements, Antibiotic resistance genes

## Abstract

**Background:**

*Vibrio cholerae* is a human intestinal pathogen and *V. cholerae* of the O139 serogroups are responsible for the current epidemic cholera in China. In this work, we reported the whole genome sequencing of a *V. cholerae* O139 strain E306 isolated from a cholera patient in the 306th Hospital of PLA, Beijing, China.

**Results:**

We obtained the draft genome of *V. cholerae* O139 strain E306 with a length of 4,161,908 bps and mean G + C content of 47.7%. Phylogenetic analysis indicated that strain E306 was very close to another O139 strain, *V. cholerae* MO10, which was isolated during the cholera outbreak in India and Bangladesh. However, unlike MO10, strain E306 harbors the El Tor-specific RS1 element with no pre-CTX prophage (VSK), very similar to those found in some *V. cholerae* O1 strains. In addition, strain E306 contains a SXT/R391 family integrative conjugative element (ICE) similar to ICE*Vch*Ind4 and SXT ^MO10^, and it carries more antibiotic resistance genes than other closest neighbors.

**Conclusions:**

The genome sequence of the *V. cholerae* O139 strain E306 and its comparative analysis with other *V. cholerae* strains we present here will provide important information for a better understanding of the pathogenicity of *V. cholerae* and their molecular mechanisms to adapt different environments.

## Background

*Vibrio cholerae* is a primary causative agent of life threatening diarrheal disease, cholera. Based on the somatic O antigens, more than two hundred serogroups of *V. cholerae* have been identified [1], among which O1 and O139 are recognized as the two major agents for cholera epidemics. *V. cholerae* serogroup O1 has two biotypes and is the causative agent for the previous two cholera pandemics, in which the classical biotype was dominant in the 6th pandemic and the El Tor in the 7th [2]. In 1992, a new non-O1 strain of *V. cholerae*, designated as serogroup O139 was identified in an epidemic cholera in India and Bangladesh [3,4]. Since then, *V. cholerae* O139 has been frequently isolated in other Asian countries where the cholera epidemics have occurred. In China, *V. cholerae* O139 strains are the dominant contributors in cholera and have been continually isolated since it first appeared in 1993 [5].

Previous studies have identified that the major virulence of *V. cholerae* O1/O139 is encoded by a lysogenic bacteriophage (CTX prophage) integrated in the *V. cholerae* genome. Many other genetic elements, such as the toxin-linked cryptic (TLC), the RS1 element, and the pre-CTX prophage (VSK), are also known to be adjacent to the CTX prophage [6]. The CTX prophage in toxigenic *V. cholerae* is usually consists of two gene clusters, the core and the RS2 regions, which are functionally different [7]. The core region includes the *ctxAB* genes encoding cholera toxin (CT), and five other genes encoding necessary components for phage morphogenesis. The RS2 region encodes proteins involved in phage replication (RstA), integration (RstB) and regulation of site-specific recombination (RstR). Another noteworthy element in *V. cholerae* is the SXT/R391 family integrative conjugative element (ICE) which was first identified in a *V. cholerae* O139 clinical isolation in 1993 [8]. The SXT/R391 ICE in *V. cholerae* usually contributes to the resistance phonotype of *V. cholerae*, encoding resistance to several antibiotics like sulfamethoxazole and trimethoprim that had previously been used for cholera treatment.

Though great efforts have been made to understand and to control this pathogen in the past, cholera caused by *V. cholerae* is still occasionally outbreak in recent years [9-11]. To date, 9 complete and nearly 200 draft genomes of *V. cholerae* are accessible in the NCBI genome projects. However, to demonstrate the evolution and the adaption mechanism of this pathogen, detailed analysis of the genomic diversity of new clinical isolations appeared in different areas and time scales is undoubtedly needed. Here, we report the genome sequence of a *V. cholerae* O139 strain E306 we recently isolated from a cholera patient in Beijing, China. The genome here will shed light on the understanding of the endemicity of cholera in North China.

## Methods

### Strain isolation

*V. cholerae* O139 strain E306 was isolated from the stool sample of a cholera case in Beijing, China, on May 30, 2013. After enrichment by alkaline peptone broth, the strain was identified as O139 serogroup by combining the results of its 16S rRNA gene sequence, serum agglutination test and biochemical reaction (Vitek 2 compact, BioMerieux Corp.). This research was approved by the Research Ethics Committee of the Institute of Microbiology, Chinese Academy of Sciences, and informed consent was obtained from the patient. The strain we reported here is available in The 306th Hospital of PLA, Beijing, China.

### Genome sequencing

The whole genome was sequenced using shotgun sequencing strategy on Illumina Genome Analyser platform. DNA Library was constructed by using the TruSeq sample preparation kit according to the manufacturer's instructions. Briefly, genomic DNA was sheared by sonication and was then end repaired. After adapters’ ligation (pair-end) with the TA cloning method, the resulting DNA fragments were size selected on a 2% agarose gel. The final DNA library was produced by PCR amplification of the selected ligation products in length of ~500 bp. DNA library (5 pM) was then loaded onto the sequencing chip; clusters were generated by using the Illumina cluster generation kit. After sequencing, image analysis and base calling were carried out by using the Illumina GA Pipeline software. Finally, a total of 6,112,322 pair-end reads were generated.

### Genome assembly and annotation

The pair-end raw sequences were quality filtered by using the DynamicTrim and LengthSort Perl scripts provided in SolexaQA suite [12]. After filtering, short reads were assembled by using SOAPdenovo (http://soap.genomics.org.cn) and the gaps were closed by using SOAP GapCloser (http://soap.genomics.org.cn). Glimmer 3.02 [13] was used for prediction of open reading frames, while tRNAscan-SE [14] and RNAmmer [15] were used for tRNA and rRNA identification, respectively. The genome was further annotated with the help of the RAST program (Rapid Annotation using Subsystem Technology) [16]. The annotation results were then checked through comparisons with the databases of NCBI-NR (http://www.ncbi.nlm.nih.gov/), COG [17], and KEGG [18]. For searching the antibiotic resistance genes, the protein-coding sequences were further Blast against Antibiotic Resistance Database (ARDB) [19], using similarity thresholds as recommended in ARDB.

### Comparative genomics

For comparative analysis, reference genome sequences of the closest genetic relatives of *V. cholerae* O139 strain E306 and representative strains belonging to important serogroups including *V. cholerae* O1 biovar El Tor str. N16961 (GenBank accession number AE003852 and AE003853), B33 (ACHZ00000000), *V. cholerae* RC9 (ACHX00000000), *V. cholerae* MO10 (AAKF03000000), *V. cholerae* MJ-1236 (CP001485 and CP001486), *V. cholerae* O1 classical O395 (CP000626 and CP000627), *V. cholerae* CIRS101 (ACVW00000000), *V. cholerae* IEC224 (CP003330 and CP003331), and *V. cholerae* O1 str. 2010EL-1786 (CP003069 and CP003070) were downloaded from the NCBI website. Whole-genome alignments and SNP identification were performed by using Progressive Mauve [20]. Concatenated SNPs in length of 23,648 bp were used to calculate the genetic distances, and a phylogenetic tree was constructed by using the neighbor-joining method in MEGA5 [21] based on these SNPs. The stability of the phylogenetic relationships was assessed by bootstrapping (1000 replicates). BWA alignment tool [22] and SAMTools [23] for SNP calling were also used for confirming the results. The genome similarities based on phylogenomic distances were analyzed using the Gegenees software [24].

### Quality assurance

The genomic DNA used for sequencing was isolated from pure culture of *V. cholerae* O139 strain E306. The 16S rRNA gene from the draft genome sequence was further confirmed to be 16S rDNA of *V. cholerae* by BLSAT against the NCBI database. Sequence contamination was also assessed by RAST annotation systems.

## Initial findings

### Genome characteristics and phylogenetic analysis

**Figure 1:**
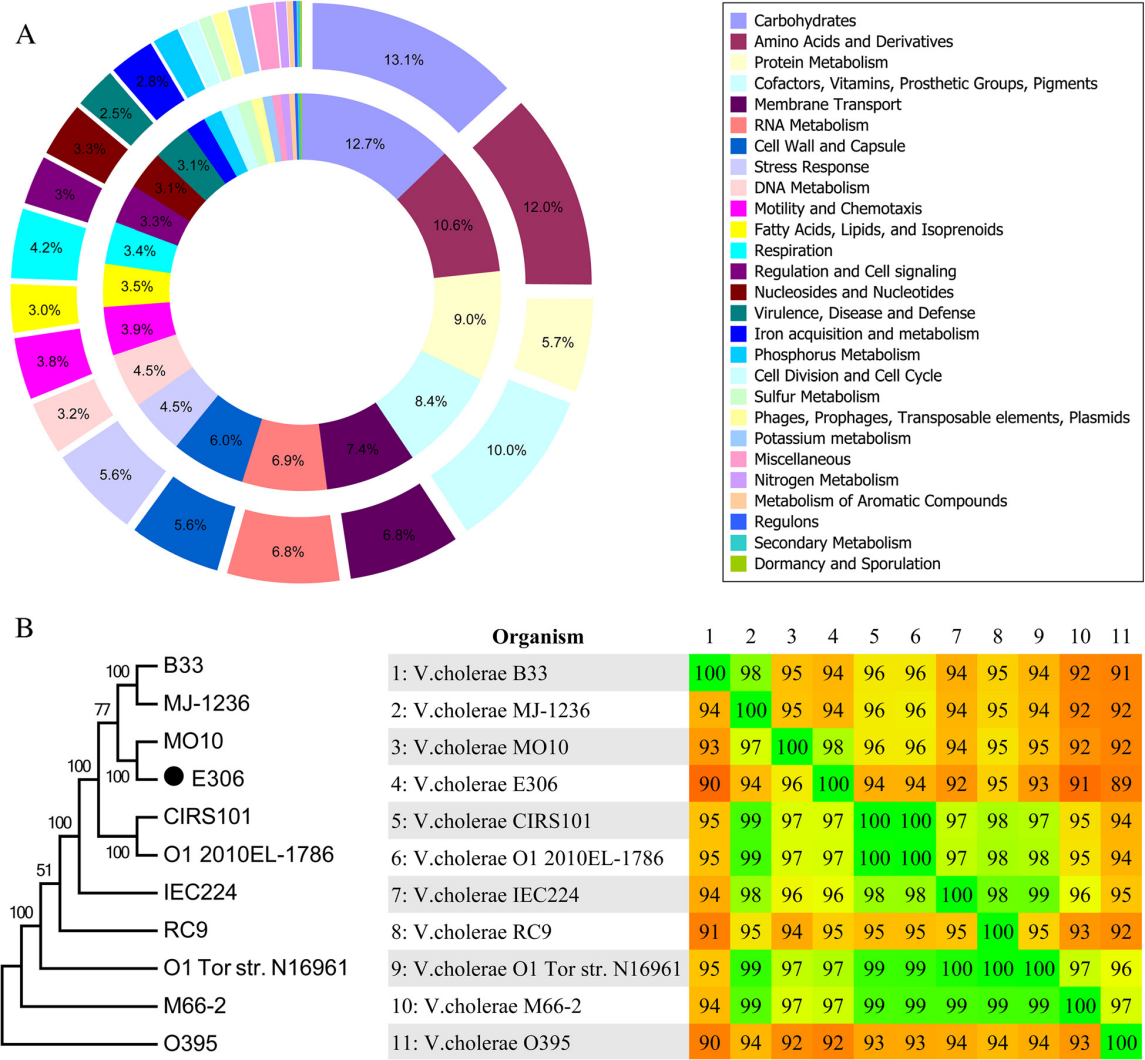
**Gene distribution and phylogenetic analysis. A.** Comparison of the distribution of genes assigned to SEED subsystems between *V. cholerae* O139 strain E306 and MO10. Outer circle and inner circle represent *V. cholerae* O139 strain E306 and MO10, respectively. Genes with less than 2% attribution are not labeled. **B.** Phylogenetic relationships (based on SNPs) of 11 *V. cholerae* strains and their genomic distance analysis. Bootstrap values less than 50% are not shown. The heat-plot of the similarity matrices is based on fragmented alignments with settings 500/500.

The genome of *V. cholerae* O139 strain E306 was sequenced on Illumina Genome Analyzer IIx platform. A total of 6,112,322 raw reads with a mean read length of 116 bp, corresponding to 170-fold coverage of the genome were generated. After assembling, a total of 51 scaffolds with N50 length of 442,144 bp were obtained, and 9 gaps were spanned by 7 scaffolds resulting in a total length of 879,788 bp. The final assembled draft genome sequence is 4,165,057 bp with mean G + C content of 47.7%. The genome contains 3861 predicted coding DNA sequences (CDSs) and 82 RNA genes (4 rRNA genes and 78 tRNA genes). RAST annotation of the whole genome indicated the presence of 534 SEED subsystems (Figure 1A). The phylogenetic tree (Figure 1B) based on whole-genome SNPs showed that the closest ancestor for O139 strain E306 was *V. cholerae* MO10, which is also a member of the O139 serogroup and was isolated during the cholera outbreak in India and Bangladesh in 1992 [3,4]. The detailed comparison of the subsystems in *V. cholerae* O139 strain E306 and *V. cholerae* O139 strain MO10 is shown in Figure 1A.

### Cholera toxin prophage

**Figure 2:**
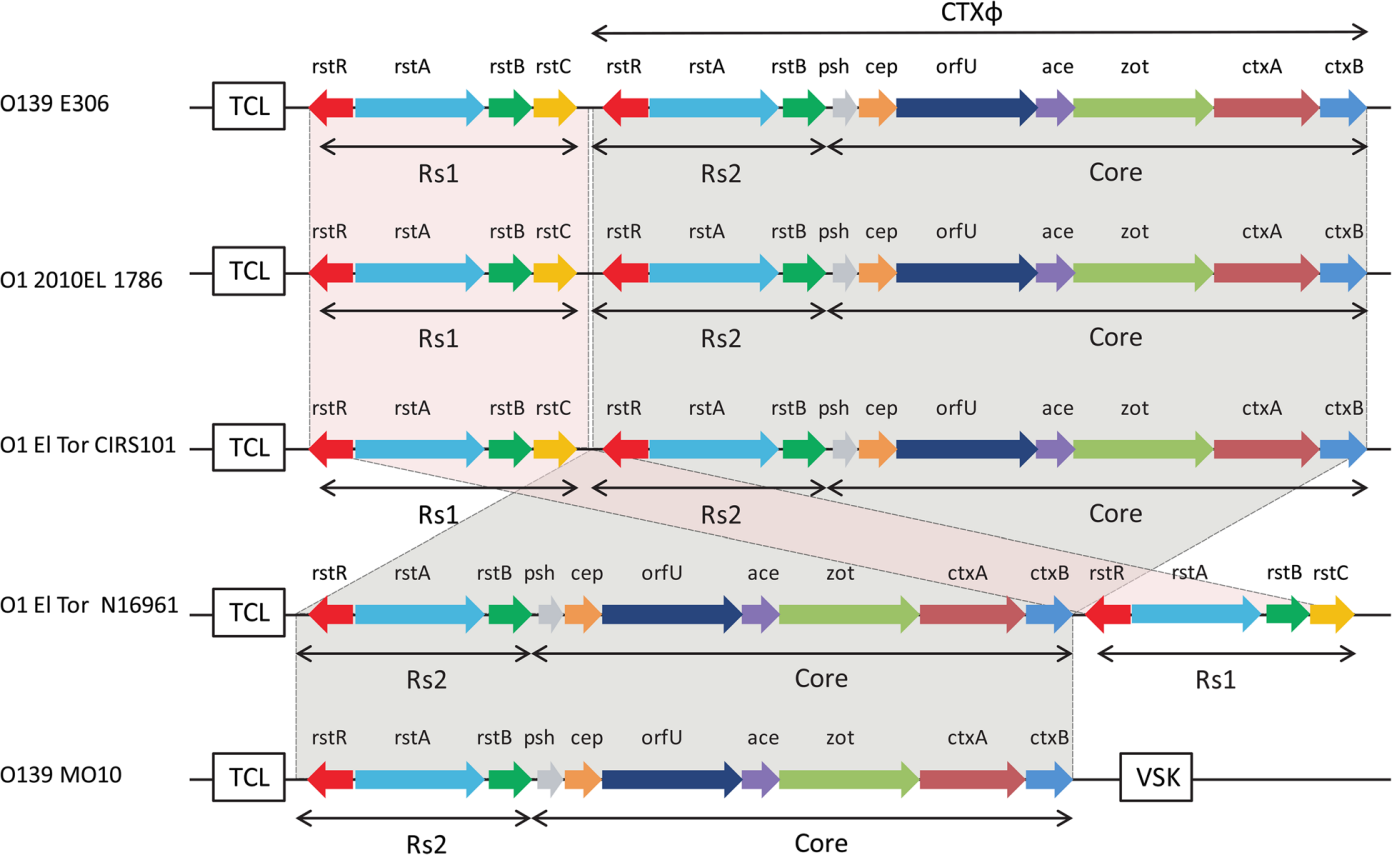
**Diagrammatic indication of the structure of the CTX prophage and associated elements in *V. cholerae *O139 strain E306 and other 4 reference strains.** The transcription direction of each gene is indicated by arrow and different genes are shaded in different colors. TLC: toxin-linked cryptic; VSK: pre-CTX prophage. The TLC and VSK elements are not drawn to scale.

Interestingly, though the *V. cholerae* O139 strain E306 is very close to *V. cholerae* O139 strain MO10, the gene organization of the cholera toxin-encoding CTX prophage is identical to those in the O1 strains of CIRS101, 2010EL-1786, and El Tor N16961. It is noteworthy that the genomic arrangements of the CTX prophage and the RS1 element in O139 strain E306, CIRS101, and 2010EL-1786 are opposite to that in *V. cholerae* O1 El Tor N16961 (Figure 2). Overall, compared with its closest neighbor, *V. cholerae* MO10, O139 strain E306 harbors the El Tor-specific RS1 element, and there is no VSK adjacent to the core region.

### Integrative conjugative elements (ICEs)

Based on the integrase gene similarity, a SXT/R391 family ICE in *V. cholerae* O139 strain E306 was identified inserted at the *prfC* locus. The general organization of this ICE was found to be highly similar to ICE*Vch*Ind4 and SXT ^MO10^ which were previously identified in *V. cholerae* O139 strains. Detailed alignment indicated that ICE^E306^ and ICEVchInd4 only differed by 3 SNPs (3 SNPs in 3 coding regions), and ICE^E306^and SXT^MO10^ differed by 26 SNPs (17 SNPs in 14 coding regions) (Table 1); no obvious large sequence changes such as deletions and insertions were observed. These results, consistent with other study [25], suggested that these ICEs in *V. cholerae* are very stable over time, and because of the high degree of similarity, the dissemination of the ICE-carrying *V. cholerae* strains between different regions cannot be excluded.

**Table 1:** **SNPs identified in SXT**^**MO10 **^**and ICE *Vch *** **Ind4 compared with ICE**^**E306**^

**Gene**	**UniProt GI**	**Product**	**Amino acid positions**	**Synonymity**	**Amino acid (codon)**		
					**ICE**^**E306**^	**SXT**^**MO10**^	**ICE *Vch *** **Ind4**
int	21885342	Integrase	326	syn	I(ATA)	I(ATT)	.
			398	NON	T(ACC)	I(ATC)	I(ATC)
rumB'	21885341	UV repair DNA polymerase	104	syn	T(ACT)	.	T(ACT)
s009	21885337	Unknown	114	NON	A(GCC)	G(GGC)	.
floR	21885343	Florfenicol exporter	230	NON	L(CTC)	V(GTC)	.
s031	21885325	Unknown	152	NON	V(GTC)	F(TTC)	.
s035	21885321	Unknown	398	NON	W(TGG)	G(GGG)	.
s037	21885319	Unknown	204	NON	P(CCG)	R(CGG)	.
s038	21885318	Unknown	26	NON	K(AAG)	R(AGG)	.
traD	21885274	Conjugative coupling factor	430	NON	D(GAC)	A(GCC)	.
			432	syn	A(GCG)	A(GCC)	.
			456	syn	V(GTG)	V(GTC)	.
s053	21885310	Unknown	194	syn	S(AGC)	S(AGT)	.
traU	21885267	Sex pilus assembly	321	NON	P(CCC)	S(TCC)	S(TCC)
s060	21885308	Unknown	194	NON	P(CCG)	T(ACG)	.
		Unknown	199	NON	S(TCA)	T(ACA)	.
setC	21885287	Transcriptional activator	20	NON	S(TCC)	Y(TAC)	.
s083	21885291	Unknown	39	NON	P(CCC)	R(CGC)	.

“.” means this position has the same amino acid and codon as ICE^E306^.

### Antibiotic resistance genes

We compared all the predicted protein-coding genes from 11 *V. cholerae* strains with known antibiotic resistance genes (BLASTp against the ARDB database [19]), yielding 50 matches to antibiotic resistance genes, mainly aminoglycoside resistance genes and tetracycline resistance genes (Table 2). A chloramphenicol resistance gene type (catb5) encoding Group B chloramphenicol acetyltransferase is present in 9 out of the 11 genomes, which is the most common resistance gene type. Interestingly, *V. cholerae* O139 strain E306 has 9 resistance genes, but no resistance gene was identified in O395 and only one was found in N16961. These results implied that different *V. cholerae* strains have different resistance profiles; the new isolation *V. cholerae* O139 strain E306 seems to have accumulated more antibiotic resistance in an environment with rapid growth rate of drug resistance [26].

**Table 2:** **Antibiotic resistance genes in the *V. cholerae *** **strains predicted by using the antibiotic resistance genes database**

**Resistance type**	**Description**	**Resistance profile**	**B33**	**CIRS101**	**IEC224**	**M66 2**	**MJ 1236**	**MO10**	**O1 2010EL**	**N16961**	**O395**	**RC9**	**E306**
ant2ia	Aminoglycoside O-nucleotidylyltransferaseadenylylation.	Tobramycin, gentamicin, dibekacin, sisomicin, kanamycin										*	
ant3ia		Spectinomycin, streptomycin	*										
aph33ib	Aminoglycoside O-phosphotransferase	Streptomycin	*	*			**	*	*			*	*
aph3ia		Paromomycin, kanamycin, neomycin, ribostamycin, lividomycin, gentamincin_b											*
aph6id		Streptomycin	*	*			*	*	*			*	*
catb5	Group B chloramphenicol acetyltransferase	Chloramphenicol	*	*	*		*	*	*	*		*	*
cml_e3	Major facilitator superfamily transporter, chloramphenicol efflux pump.	Chloramphenicol		*			*	*	*				*
dfra1	Group A drug-insensitive dihydrofolate reductase	Trimethoprim					*		*				
dfra23		Trimethoprim										*	
sul1	Sulfonamide-resistant dihydropteroate synthase	Sulfonamide	*									*	*
sul2		Sulfonamide	*	*			*	*	*			*	*
teta	Major facilitator superfamily transporter, tetracycline efflux pump	Tetracycline					*						
tetc		Tetracycline						*					
tetd		Tetracycline										*	*
tetm	Ribosomal protection protein	Tetracycline											*

Stars mean the number of antibiotic resistance genes found.

## Future directions

Compared to the epidemic lineages of *V. cholerae* serogroup O1, our understanding of the genomic properties and their diversity of *V. cholerae* serogroup O139 is very limited. In this study, we sequenced the whole genome of a newly isolated strain of *V. cholerae* O139. This strain, carrying an El Tor-specific RS1 element that was found in *V. cholerae* O1 serogroup and more antibiotic resistance genes than other sequenced strains, highlights its high ability to adapt to new environments and poses a risk of causing new epidemic cholera. Moreover, the genome here will be of great interests for future *V. cholerae* comparative genomics.

## Availability of supporting data

This Whole Genome Shotgun project has been deposited at DDBJ/EMBL/GenBank under the accession AWWA00000000. The version described in this paper is version AWWA01000000.

## Competing interests

The authors declare that they have no competing interests.

## Authors’ contributions

YY and FL interpreted the sequencing data and prepared the manuscript. NL, JL and RFZ generated the sequencing data. YFH participated all discussions of data analysis and rewrite the manuscript. YFH, YY, BLZ and YC were involved in overall experimental design. All authors have read the manuscript and approved.
